# Correlation Analysis of Expression Profile and Quantitative iTRAQ-LC-MS/MS Proteomics Reveals Resistance Mechanism Against TuMV in Chinese Cabbage (*Brassica rapa* ssp. *pekinensis*)

**DOI:** 10.3389/fgene.2020.00963

**Published:** 2020-08-20

**Authors:** Shanwu Lyu, Liwei Gao, Rujia Zhang, Changwei Zhang, Xilin Hou

**Affiliations:** ^1^State Key Laboratory of Crop Genetics and Germplasm Enhancement, Nanjing Agricultural University, Nanjing, China; ^2^College of Horticulture, Nanjing Agricultural University, Nanjing, China

**Keywords:** transcriptome (RNA-Seq), proteome, correlation analysis, Chinese cabbage, *Turnip mosaic virus* (TuMV) resistance

## Abstract

The arms race between plants and viruses never ceases. Chinese cabbage, an important type of *Brassica* vegetable crop, is vulnerable to plant virus infection, especially to *Turnip mosaic virus* (TuMV). To better examine the molecular mechanisms behind the virus infection, we conducted the correlation analysis of RNA-Seq and quantitative iTRAQ-LC-MS/MS in TuMV-infected and in healthy Chinese cabbage leaves. There were 757 differentially expressed genes and 75 differentially expressed proteins that were screened in Chinese cabbage plants infected with TuMV. These genes were enriched in many pathways, and among them, the plant hormone signal transduction, plant-pathogen interaction, and protein processing in the endoplasmic reticulum pathways were suggested to be closely related pathways. The correlation analysis between RNA-Seq and quantitative iTRAQ-LC-MS/MS was then further explored. Finally, we obtained a preliminary network of several candidate genes associated with TuMV infection, and we found that they mainly belonged to calcium signaling pathways, heat shock proteins, WRKY transcription factors, and non-specific lipid transfer proteins. These results may lead to a better understanding of antiviral mechanisms and of disease-resistant breeding.

## Introduction

*Brassica* vegetable crops not only provide multiple components of nutrition such as proteins, vitamins, trace elements, and inorganic salts, but also serve as important research materials. Among *Brassica* vegetable crops, Chinese cabbage (*Brassica rapa* ssp. *pekinensis*) has the largest cultivated area and yield, and the completion of its genome sequence in 2011 has made its transcriptomic and proteomic analysis more feasible (Cao et al., 2006; Cheng et al., 2011).

However, several biotic stresses have been constant threats to both crop quality and yield. *Turnip mosaic virus* (TuMV), a member of the *Potyvirus* genus, has emerged as an important pathogen to *Brassica* crops (Walsh and Jenner, 2002). It ranks only second to *Cucumber mosaic virus* (CMV) and is the most prevalent viral pathogen infecting Chinese cabbage, especially in Asia, North America, and Europe (Tomlinson, 1987). It infects 318 kinds of dicotyledons and several monocotyledons through artificial inoculation (Walsh and Jenner, 2002; Shattuck, 2010). Moreover, it can develop conspicuous symptoms such as systemic vein clearing, necrosis, chlorosis, and withering; internal necrosis would occur following further cold storage (Li et al., 2019). On account of its broad parasitifer and its non-persistent transmission, many means of prevention against TuMV are ineffective, and some of them can cause agricultural chemical pollution (Walsh and Jenner, 2002). Furthermore, climate change also enhances the difficulties in controlling damage caused by plant viruses (Jones and Barbetti, 2012). As such, investigation of natural plant genes that confer resistance against viruses may provide effective and environmentally friendly ways for the reduction or restriction of viral infections (Fraser, 1990; Hughes et al., 2002; Walsh and Jenner, 2002; Li et al., 2019).

Plant viruses are a type of obligate intracellular parasite, so their infection cycles are intimately related to and rely completely on the host cell component, including host factors. Host factors encompass proteins, nucleic acids, carbohydrates, lipids, and metabolites which can be used for fighting off pathogens (Laliberte and Sanfacon, 2010). Accordingly, the discovery of new TuMV host factors and their applications in production using modern molecular biology techniques are a trend of modern genetic engineering to confer plant disease resistance. Up to now, several TuMV resistance loci or genes have been mapped or cloned in *Brassica* vegetable crops. In *Brassica napus*, *TuRB01* and *TuRB02* were initially mapped (Walsh et al., 1999), afterward, *TuRB04* and *TuRB05* were identified by Jenner et al. (2002, 2003), followed by *TuRB03* (Hughes et al., 2003). In *Brassica rapa*, *ConTR01*, *retr01* (Rushholme, 2000; Walsh and Jenner, 2002; Rusholme et al., 2007), *BcTuR3* (Ma et al., 2010), *Rnt1-1* (Fujiwara et al., 2011), *TuRBCH01* (Xinhua et al., 2011), *retr02* (Qian et al., 2013), and *TuRB01b* (Lydiate et al., 2014), have been identified, as well as *retr03* (Shopan et al., 2017) in *Brassica juncea*. Moreover, numerous disease resistance (*R*) genes also have been identified including the NBS-LRR gene family. Several genes in this family have been reported such as *HRT*, *N*, *RCY1*, *Rx1*, *Rx2*, and *Sw5* (Kang et al., 2005). Aside from the *R* genes, the translation initiation factors *eIF4E* and *eIF4G* were also isolated as recessive resistance genes in *Arabidopsis thaliana* and *B. rapa* (Sanfaçon, 2015; Li et al., 2018). Recently, *retr02* has been suggested to be an allele of *eIF(iso)4E* (Qian et al., 2013), and that eIF4E-related resistance has been reported as the more effective type (Yeam et al., 2007; Mazier et al., 2011; Rodríguez-Hernández et al., 2012). Chung et al. (2014) constructed a genetic map based on high-throughput SNP and discovered a novel dominant TuMV resistance locus (*TuMV-R*), containing four CC-NBS-LRR resistance genes and two pathogenesis-related-1 genes. Recently, Wang et al. (2015) identified TuMV-responsive as well as new types of miRNA in non-heading Chinese cabbage through high-throughput sequencing.

Despite these excellent signs of progress, functional data remain scarce, especially in terms of finding new host factors involved in virus infection, replication, or transport. To this end, we conducted the correlation analysis by combing RNA-Seq and quantitative iTRAQ-LC-MS/MS on Chinese cabbage infected with TuMV. Most of the differentially expressed genes (DEGs) and differentially expressed proteins (DEPs) belong to the plant hormone signal transduction, plant-pathogen interaction, and protein processing in the endoplasmic reticulum pathways. Subsequently, we found many candidate resistance genes associated with TuMV infection, mainly focusing on the calcium signaling pathways, heat shock proteins, WRKY transcription factors, and lipid transfer proteins. These results can serve as an important source in research involving antiviral mechanisms and disease-resistant breeding.

## Materials and Methods

### Plant Material

The Chinese cabbage (*chiifu-401-42*) used in this study is a typically sequenced cultivar and medium sensitive to TuMV infection. Seeds were pre-germinated for 2 days at 25°C and were transferred into an incubator set at 20–25°C with a 16-h light/8-h dark photoperiod and a constant 85% relative humidity. Mechanical inoculation with TuMV was performed based on a previous report (Lv et al., 2015). Briefly, the five-leaf stage seedlings were rubbed with the TuMV inoculum (pathotype C4) by grounding the TuMV-infected Chinese cabbage leaves with phosphate buffer (pH 7.4). The plants inoculated only with buffer served as control. At 21 days post-inoculation (dpi), >2.0 g each of the infected and the control leaves were collected for RNA-Seq, proteomics, and qRT-PCR verification. After collection, all samples were immediately frozen using liquid nitrogen and were stored at −80°C for further experiments. Three biological replicates were conducted for each of the subsequent analysis.

### RNA Isolation and Sequencing

For Illumina sequencing, total RNA was extracted from the TuMV-infected and control leaves using TRIzol reagent (Takara Bio Inc., Otsu, Japan) following the manufacturer’s protocol. RNA quantity and quality assessment were performed using the Agilent 2100 Bioanalyzer (Agilent, Santa Clara, CA, United States) and gel electrophoresis. After treatment with DNase I and enrichment using the oligo (dT) magnetic beads (for eukaryotes), the mRNA was fragmented into short fragments (about 200 bp). Subsequently, double-stranded cDNA was synthesized, and the sequence adaptors were ligated to the fragments. Finally, the Agilent 2100 Bioanalyzer and ABI StepOnePlus Real-Time PCR System were used to qualify and quantify the sample libraries. These libraries were sequenced at the Beijing Genomics Institute (Shenzhen, China^1^) using the Illumina HiSeq^TM^ 2000 (San Diego, CA, United States).

### Quantitative Real-Time PCR Validation and RT-PCR Detection

The RNA of each sample was extracted as previously described and was reverse-transcribed using the PrimeScript^TM^ RT reagent Kit with gDNA Eraser (Perfect Real Time Takara Bio Inc., Otsu, Japan). For RT-PCR, the amplification procedure used was as follows: initial denaturation at 94°C for 4 min, followed by 30 cycles of 94°C for 30 s, 55°C for 30 s, and 72°C for 30 s. Final extension was performed at 72°C for 10 min. The length of the coat protein (*CP*; GenBank: EF028235) gene segment is 204 bp. To verify the reliability of Illumina sequencing, we randomly selected 25 genes from the 757 DEGs. Their transcript levels were quantified using the 2^–ΔΔCT^ method (Livak and Schmittgen, 2001). qRT-PCR was performed in three biological replicates following protocols used in the previous study (Lv et al., 2015). All of the specific primers for the 25 genes were designed using Beacon Designer v 7.9, and Chinese cabbage *Actin* (*Bra028615*) was used as the reference gene for quantitative expression analysis (Supplementary Table S1). Correlation test of RNA-Seq and qRT-PCR was analyzed by using ggpubr (v 0.4.0) R package^2^.

### Protein Extraction and Peptide iTRAQ Labeling

Total proteins were extracted from the TuMV-infected and control leaves. Samples were homogenized in lysis buffer solution (7 M Urea, 1 mM PMSF, 2 mM EDTA, 2 M Thiourea, 10 mM DTT, and 4% CHAPS) and were precipitated by acetone. Then, 100 μg digested proteins of each sample were labeled with different isobaric tags, and the iTRAQ tags used were as follows: CK_1-114 isobaric tag, CK_2-116 isobaric tag, CK_3-118 isobaric tag, TuMV_1-117, TuMV_2-119, and TuMV_3-121. These samples were blended and lyophilized before conducting strong cation exchange chromatography (SCX) using the Shimadzu LC-20AB HPLC system (Shimadzu, Kyoto, Japan). Each of the above components was fractionated using a Prominence LC-20AD Nano HPLC (Shimadzu, Kyoto, Japan). Finally, Q-EXACTIVE (Thermo Fisher Scientific, San Jose, CA, United States), which involves nanoelectrospray ionization followed by tandem mass spectrometry (MS/MS), was used for data acquisition. The resolution used in the Orbitrap was 70,000, and the higher-energy collision dissociation (HCD) mode (27 ± 12% collision energy) was used for MS/MS.

### Bioinformatics Analysis

The Brassicaceae Genome Data v 1.5^3^ was used as the reference genome. After removing data corresponding to impurities using the Trimmomatic program (v 0.33) (Kim et al., 2018), clean reads from the raw data were mapped to the reference genome using a short read alignment software SOAPaligner/SOAP2 (Li et al., 2009) with two mismatches for mapping the reads to the reference genome. The result mapped to the genome was recorded for further analysis. Expression levels of each unigene were calculated using the reads kilobases per million reads (RPKM) method (Mortazavi et al., 2008). Afterward, we used the Pearson correlation coefficients (PCC) to assess repeatability within replications. The longest transcript was used for the calculation of gene expression and coverage if there were several transcripts in a unigene. We used a false discovery rate (FDR) of ≤0.001 and the absolute value of log_2_Ratio ≥ 1 to screen for DEGs. Mascot v2.3.02 was used for protein identification against the database containing Bra_Chromosome_V1.5 (40796 sequences)^4^. Ratios with *p*-values < 0.05 and fold changes > 1.2 were considered as significant. Functional annotations were conducted using the Blast2GO program against the non-redundant protein database (NR; NCBI), KEGG database^5^ and the COG database^6^.

## Results

### Symptom and Molecular Identification of TuMV-Infected Chinese Cabbage

**FIGURE 1:**
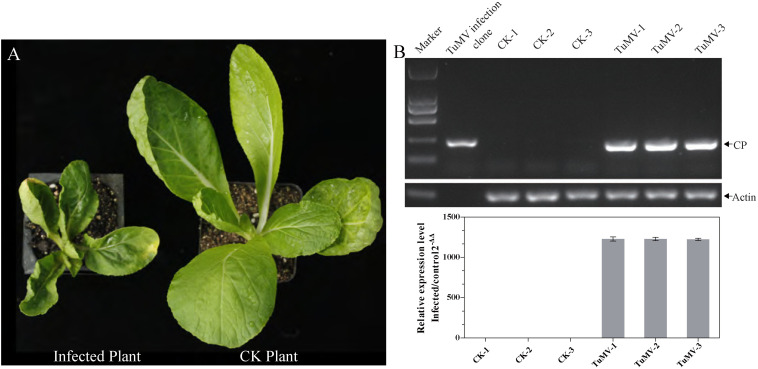
The identification of morbidity in inoculated Chinese cabbage. **(A)** The left plant was photographed at 21 dpi, and it showed severe symptoms such as systemic vein clearing, necrosis, and stunting. The right plant served as control. **(B)** For RT-PCR detection, RNA extraction and reverse transcription were performed on each sample. *CP* expression level was quantified by qRT-PCR, and the length of *CP* segment was 204 bp. *Actin 7* (*Bra028615*) was used as an internal reference gene.

Before sampling, we first determined whether all the inoculated Chinese cabbage were infected by TuMV. The phenotypes and transcription levels were used as evaluating indicators. Results showed that all the inoculated Chinese cabbage presented severe symptoms such as severe systemic vein clearing, necrosis, and stunting (Figure 1A). Upon obtaining the RT-PCR and qRT-PCR results, the 204 bp amplicons were observed to be quite clear in the infected samples, compared with the healthy ones, and TuMV was observed to have largely proliferated (average relative expression level = 1226.30, Figure 1B). The results indicated the presence of the TuMV virion in inoculated samples and showed that the appropriate time for sampling is 21 dpi.

### RNA Sequencing, Mapping, and DEGs Screening

**FIGURE 2:**
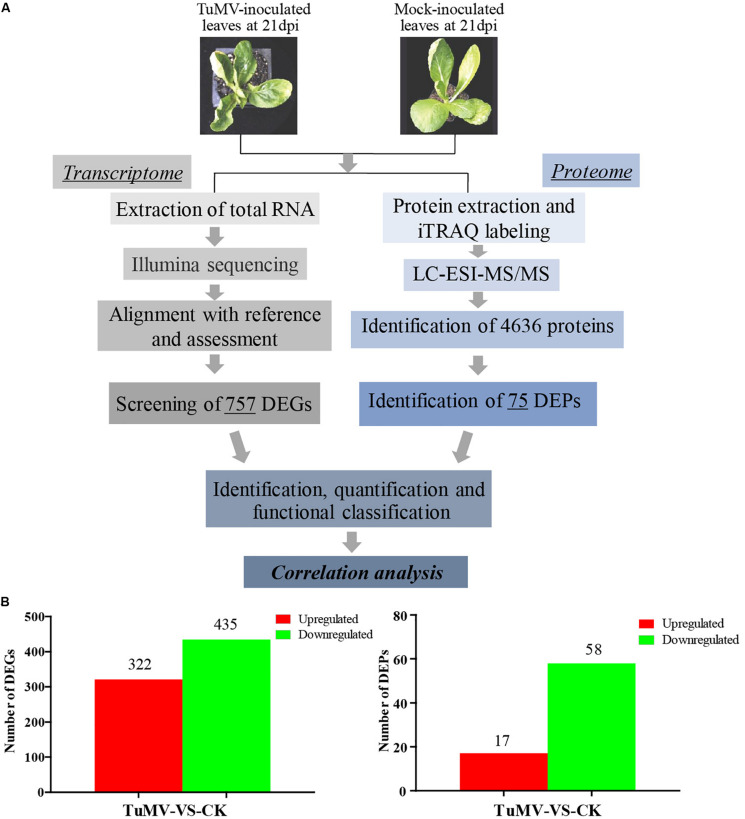
Experimental workflow and summary of the present study. **(A)** Transcriptomic and proteomic methods that were used to analyze correlation and to find viral disease-related genes. **(B)** Significant differentially expressed genes screened at the transcription and proteome levels.

The expression levels of the transcriptome and proteome were detected by RNA-Seq and iTRAQ according to the workflow illustrated in Figure 2A. The results of transcriptome, proteome, and correlation analysis are illustrated in Table 1. A standard bioinformatics analysis was performed as follows: first, after deleting low-quality reads and adaptor sequences, we obtained 7,195,399 (CK_1, 59.83%), 7,610,342 (CK_2, 64.63%), 8,119,719 (CK_3, 65.55%), 6,486,095 (TuMV_1, 55.35%), 7,563,676 (TuMV_2, 62.89%), and 7,503,701 (TuMV_3, 62.25%) mapped reads in total, compared with the total reads, corresponding to 12,025,649, 11,775,794, 12,387,122, 11,718,227, 12,026,610, and 12,054,525 based on the Brassicaceae Genome Data v 1.5 (Supplementary Table S2). The average value of unique matches was 58.12% with a 17.70% mismatch (≤2 bp). Then, we performed a quality assessment of reads, and the average clean reads accounted for 99.43%. Standard deviations (SD) between the biological replicates were 0.1% (CK) and 0.05% (TuMV), respectively (Supplementary Table S3 and Supplementary Figure S1), indicating that the sequencing results were of high quality and were reproducible. Next, to determine sequencing depth, our analysis results showed that the number of identified genes became saturated when the number of reads reached 12 million. In our study, the number of each sample exceeded 6 million (Supplementary Figure S2). Sequencing libraries were built according to the method reported by Wang et al. (2009), which led to an increased randomness (Supplementary Figure S3). Gene coverage is also an important assessment index of sequencing quality. Here, more than 50% of genes corresponded to over 80% of the gene coverage (Supplementary Figure S4). Finally, in the statistical analysis of gene expression levels, we applied the RPKM method to estimate transcript abundance, as well as all the other information, including gene length, RPKM, gene coverage, log_2_ratio (TuMV/CK), and probability; these are shown in Supplementary Table S4.

**TABLE 1:** Summary of results of the transcriptome, proteome, and correlation analysis.

Group name	Type	Number of proteins	Number of genes	Number of correlations
TuMV-VS-CK	Identification	4636	31513	4592
TuMV-VS-CK	Quantitation	3498	31513	3476
TuMV-VS-CK	Differential Expression	75	757	12

Moreover, to screen DEGs, we used the NOIseq method (Tarazona et al., 2011) according to its good true positive and false-positive rates. Finally, we obtained 757 remarkable DEGs following a filtering condition of fold change ≥ 2 and probability ≥ 0.8 (Figure 2B and Supplementary Table S5). Among them, 322 genes were found to be upregulated and 435 genes were downregulated.

### qRT-PCR Based Validation

**FIGURE 3:**
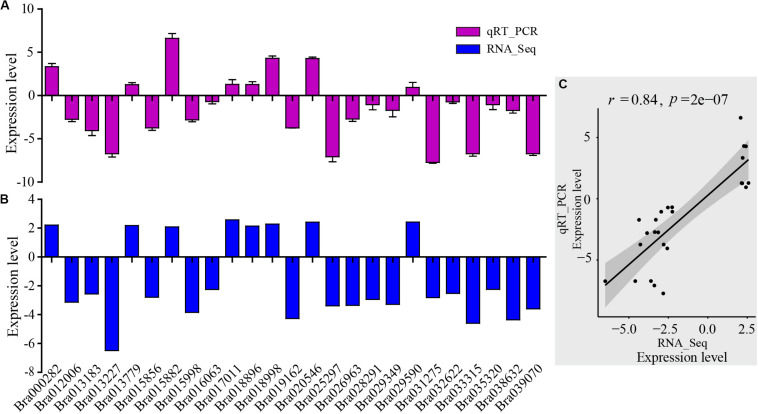
Validation of RNA-Seq results by qRT-PCR. **(A)** qRT-PCR expression levels were determined based on the blue bar. **(B)** RNA-Seq expression levels were determined based on the purple bar. **(C)** Scatter plots of correlation test between qRT-PCR and RNA-Seq results. *r* is Pearson correlation coefficient. The *p*-value is the significance level of the correlation. There were 25 genes randomly selected from the DEGs related to AP2-EREBP transcription factors, basic helix-loop-helix (bHLH) transcription factors, chloroplast and mitochondria gene families, cytochrome P450, MAP kinase (MAPK) families, MYB gene families, NAC transcription factor family, and WRKY transcription factor family.

For assessment of the RNA-Seq data, RNA-Seq libraries were subjected to qRT-PCR. First, 25 genes were selected randomly from the DEGs which were related to the AP2-EREBP transcription factors, basic helix-loop-helix (bHLH) transcription factors, chloroplast and mitochondria gene families, cytochrome P450, MAP kinase (MAPK) families, MYB gene families, NAC transcription factor families, and WRKY transcription factor families. The qRT-PCR results of these metabolism and disease resistance genes coincided with the RNA-Seq data (*r* = 0.84, *p*-value = 2e-07), demonstrating the high reliability of RNA-Seq data (Figure 3).

### Annotation and Functional Classification

For an improved understanding of the gene functions, all the identified unigenes and proteins, DEGs, and DEPs were annotated by aligning the Nr database of NCBI to GO terms using BLAST2GO (default parameters) (Conesa et al., 2005). Enrichment was performed by WEOGO (Ye et al., 2006). Genome-wide transcriptome or proteome expression levels were used as background.

**FIGURE 4:**
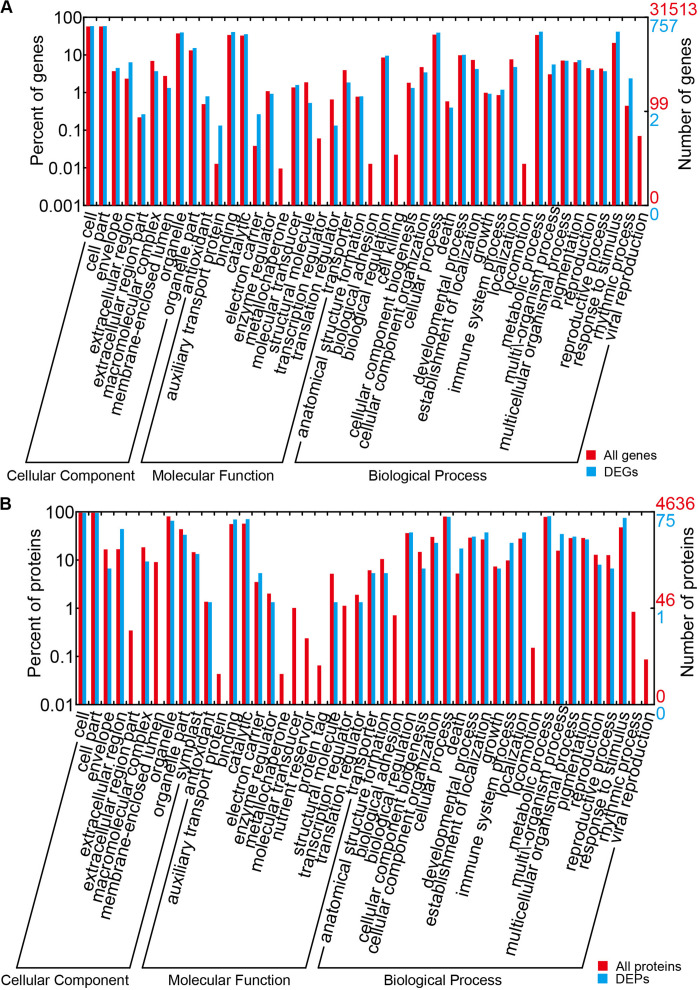
GO term assignment of DEGs and DEPs in Chinese cabbage. **(A)** GO categories for DEGs in the transcriptome. **(B)** GO categories for DEPs in the proteome. The whole transcriptome or proteome was used as the background. Results were summarized for the three main GO categories: biological process (P), molecular function (F), and cellular component (C).

The results of the DEG GO reassignments showed that all unigenes were categorized into 44 GO terms in three ontologies, consisting of biological process (23 subcategories), cellular component (9 subcategories), and molecular function (12 subcategories) (Figure 4A and Supplementary Table S6). In biological process, ‘percentages of genes in the rhythmic process,’ ‘response to stimulus process,’ ‘multi-organism process,’ ‘metabolic process,’ ‘immune system process,’ ‘biological process,’ and ‘cellular process’ were higher than the genome-wide transcriptome expression levels. In the classification of biological process, it was found that this category exerted repercussions on TuMV infection. In molecular function, ‘molecular function transporter activity’ and ‘binding’ were the two predominant subcategories, and the proportion of ‘auxiliary transport protein’ in the DEGs was significantly higher than that of the background. This suggested that more auxiliary transport proteins were involved in the process of TuMV infection. In the cellular component, the ‘extracellular’ and ‘extracellular region’ were the dominant subcategories. There was no significant difference between DEGs and genome-wide genes in this part.

**FIGURE 5:**
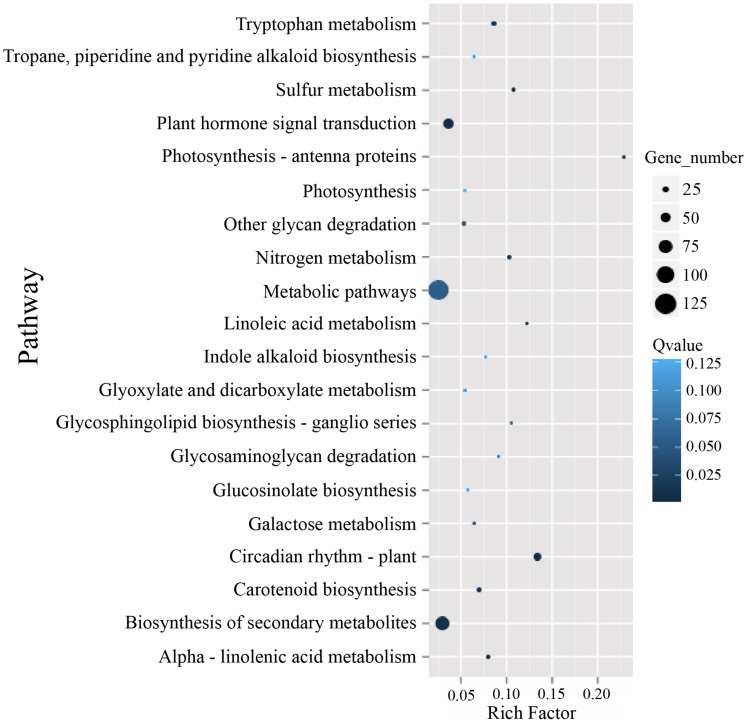
The KEGG enrichment scatterplot of the top 20 statistically significant pathways plotted for the DEGs. The rich factor on the horizontal refers to the ratio between the gene numbers of DEGs which are located at the pathway entry as well as the total gene numbers of all annotated genes which are located at the pathway entry. *Q*-value was determined by multiple hypothesis testing corrections, and their values vary from 0 to 1.

To identify possible pathways related to TuMV infection, all the unigenes were annotated by the Kyoto Encyclopedia of Genes and Genomes (KEGG) database^5^ (Kanehisa et al., 2007) (Supplementary Table S7), and the pathways with *Q* value ≤ 0.05 were considered as significantly enriched. The top 20 enrichment terms are shown in Figure 5. The majority of these pathways are metabolic pathways (125/4990 genes), biosynthesis of secondary metabolites (81/2757 genes), plant hormone signal transduction (56/1555 genes), circadian rhythm (36/269 genes), and plant-pathogen interaction (34/1633 genes) pathways. In addition, protein processing in the endoplasmic reticulum (10/610 genes), regulation of autophagy (6/156 genes), and endocytosis (4/265 genes) may be involved in TuMV assembly and transport.

### Proteomics Characterized by iTRAQ and Data Analysis

The Mascot search engine (Matrix Science, London, United Kingdom; version 2.3.02) was used in protein identification against the Bra_Chromosome_V1.5 database containing 40,796 sequences. The quality error distribution of spectrogram matching was conducted to assess the identifying quality (Supplementary Figure S5A). 92,152 spectra out of 366,867 total spectra were matched after quality control, and among them, 60,115 were considered as unique spectrums. A total of 13,348 unique peptides were identified, and 4,636 proteins were obtained (Supplementary Figure S5B). The protein mass distribution ranged from 7.608 to 592.995 kDa, corresponding to *Bra005963* and *Bra013017*, and the percentage of >20 kDa accounted for 93% (4316/4636) (Supplementary Figure S5C). Since stable results can be obtained based on the stability of the mass spectrum experiment process, we conducted repeated analysis (Supplementary Figure S5D). The obtained mean coefficient of variation (CV) was 9.6%, suggesting that the data were reliable for further analysis. For protein abundance, at least two unique spectra contained in a protein was set as the standard. Proteins with *p*-values < 0.05 and fold changes of > 1.2 were considered as DEPs (Supplementary Figure S5E). Under these thresholds, a total of 75 DEPs were obtained, while 17 proteins were upregulated and 58 were downregulated (Figure 2B and Supplementary Table S8).

The results of GO reassignments showed that proteins were also categorized into three ontologies (Figure 4B and Supplementary Table S9); however, compared with the all identified proteins, ratios of ‘response to stimulus,’ ‘multi-organism process,’ ‘immune system process,’ and ‘death’ in ‘biological process’ were slightly higher. Compared with the DEG GO results, these were all high in ‘response to stimulus,’ ‘multi-organism process,’ and ‘immune system process.’ In the molecular function component, ‘binding and catalytic’ were high, but no gene was detected in ‘auxiliary transport protein.’ In the cellular component, ‘extracellular region’ was the only term in which the significant protein ratios were higher than that of all identified proteins. All the DEPs were also annotated to 45 pathways by retrieving the KEGG database (Supplementary Table S10). There were seven pathways that contained greater than or equal to four proteins, namely, metabolic pathways (17, 32.08%), biosynthesis of secondary metabolites (11, 20.75%), nitrogen metabolism (5, 9.43%), pyruvate metabolism (5, 9.43%), arginine and proline metabolism (4, 7.55%), plant hormone signal transduction (4, 7.55%), and plant–pathogen interaction (4, 7.55%).

### Correlation Analysis of Transcriptome and Proteome

To explore the congruence of fold change between the transcriptome and proteome data, we conducted correlation analysis. The quantitative relation of the proteins and genes are shown in Table 2. The filter criteria of proteins and genes used was fold change ≥ 1.2, *p*-value ≤ 0.05, fold change ≥ 2, and FDR ≤ 0.001.

**TABLE 2:** Correlation analysis between the transcriptome and proteome.

Protein ID	Quantitation (TuMV/CK)	Sig	Diff Protein	Gene ID	log2(TuMV/CK)	Probability	Diff gene
**Same trend**							
Bra016926	1.28	*	+	Bra016926	1.57	0.84	+
Bra020322	1.78	*	+	Bra020322	1.63	0.86	+
Bra022468	0.75	*	–	Bra022468	−1.29	0.80	–
Bra006721	1.54	*	+	Bra006721	2.39	0.90	+
**Different trend**							
Bra028091	0.64	*	–	Bra028091	2.05	0.88	+
Bra000876	0.60	*	–	Bra000876	2.32	0.90	+
Bra025730	0.57	*	–	Bra025730	2.25	0.90	+
Bra003273	0.69	*	–	Bra003273	1.58	0.85	+
Bra000315	0.71	*	–	Bra000315	1.29	0.82	+
Bra036259	0.78	*	–	Bra036259	3.38	0.83	+
Bra004771	0.53	*	–	Bra004771	2.79	0.90	+
Bra015656	1.24	*	+	Bra015656	–1.22	0.81	–

*: Significance; +: upregulated; −: downregulated.

**FIGURE 6:**
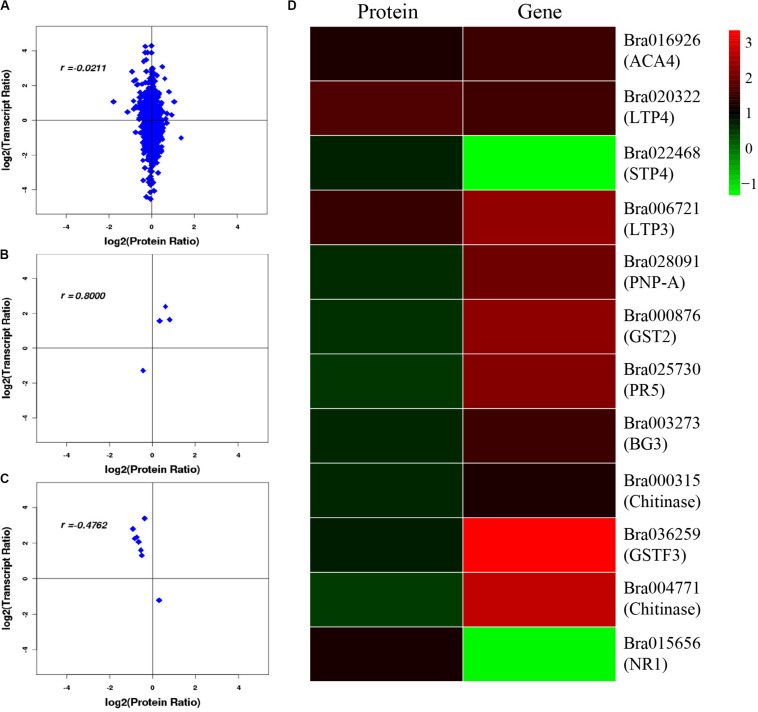
Correlation analysis between transcriptome and proteome. **(A)** Correlation analysis of all DEGs and DEPs. **(B)** Correlation analysis of DEGs and DEPs with similar changing trends. **(C)** Correlation analysis of DEGs and DEPs with opposite changing trends. **(D)** Heatmap for 12 pairs of DEGs and DEPs. Red represents upregulated genes and green represents downregulated genes. Gene ID and gene names were attached on the right. ACA4: Autoinhibited Ca(2+)-atpase, isoform 4, LTP4: Lipid transfer protein 4, STP4: Sugar transporter 4, LTP3: Lipid transfer protein 3, PNP-A: Plant natriuretic peptide A, GST2: Glutathione *s*-transferase phi 2, PR5: Pathogenesis-related protein 5, PR2/BG2: Pathogenesis-related protein 2/beta-1,3-glucanase 2, Chitinase: Chitinase family protein, GSTF3: Glutathione s-transferase f3, Chitinase: Chitinase, NR1: Nitrate reductase 1. For the correlation analysis diagram **(B–D)**, the levels of the DEPs were labeled on the horizontal axis, and the levels of the DEGs were labeled on the vertical.

After correlation analysis of the expression data at the proteomic and at the transcriptional level, we found that correlation between the two was low (*R* = −0.0211) (Figure 6A and Supplementary Table S11), and that the genes that can be detected in the proteome but not in the transcriptome and those that were detected in the transcriptome, were higher (Supplementary Tables S12, S13). This inferior correlation between the mRNA and protein abundance ratios showed the inconsistencies between transcription and translation in the plant system; moreover, temporal and spatial discrepancies in the function and the complexity of biological regulatory networks can also explain this result. Although the overall correlation degree was low, there were also a few genes that had a high correlation with the proteins. *Bra016926*, *Bra020322*, *Bra022468*, and *Bra006721* have the same trend in the changes of the *R* value = 0.8000 (Figures 6B,D and Table 2). Additionally, there were eight genes (*Bra028091*, *Bra000876*, *Bra025730*, *Bra003273*, *Bra000315*, *Bra036259*, *Bra004771*, and *Bra015656*) that showed an opposite change trend, with *R* value = −0.4762 (Figures 6C,D and Table 2).

### Transcriptome and Proteome Data Mining

For further analysis, DEGs were then mapped to their predicted pathways (Supplementary Table S7). A total of 56 genes were mapped to the plant hormone signal transduction pathway. In this group, genes related to auxin were the majority, including Gretchen Hagen 3 (GH3) family genes and small auxin upregulated RNAs (SAUR) family genes. A clear majority of genes in this group were significantly downregulated. The cytokinin signal pathway-related genes ranked second, containing three cytokinin receptors and seven *Arabidopsis* response regulator (ARR) family genes. Most genes in this group were upregulated. The last major group was related to jasmonate (jasmonate ZIM domain-containing protein), and all the genes in this group were downregulated. Moreover, 34 genes were mapped to the plant–pathogen interaction pathway. The group of jasmonate related genes were also present in the hormone signal transduction pathway. There were five LRR receptor-like serine/threonine-protein kinase-related genes, and three of these were downregulated and two were upregulated. There were five WRKY transcription factors and only one was downregulated. As protein processing in the endoplasmic reticulum is important for virus reproduction and assembly, we also chose to focus on this pathway. In this group, heat shock proteins (HSPs) were the majority, and most of the HSPs belonged to HSP20. In addition, we also mapped six genes to the regulation of autophagy pathway, and they were all downregulated. Since virus replication mainly occurs in the intracellular membrane structure, the genes in the protein processing in endoplasmic reticulum pathway may be related to virus replication. Eight out of ten genes in this group belonged to the HSPs. As previously reported, the autophagy pathways are related to *Potyvirus* infection (Cheng and Wang, 2017), thus, we also paid attention to this pathway. We found that it consisted of six genes and that all of them were downregulated.

**FIGURE 7:**
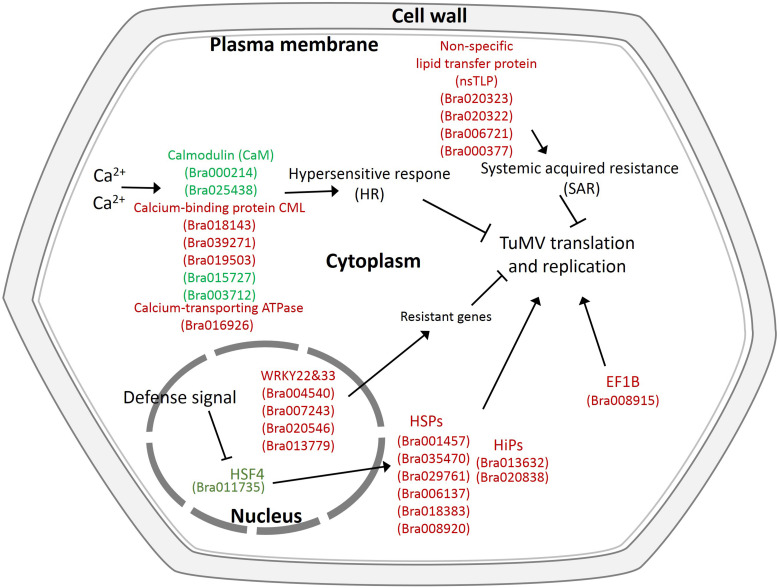
Proposed model depicting related networks in Chinese cabbage under TuMV infection. The majority of the significant differentially expressed genes or proteins related to TuMV infection are shown in color. Red denotes upregulated genes or proteins, and green represents downregulated genes or proteins.

In addition, there were also many disease-related genes found in the DEPs. In the upregulated proteins, there were four non-specific lipid transfer proteins (nsLTPs) (Bra020323, Bra020322, Bra006721, and Bra000377) that were predicted to encode pathogenesis-related (PR) proteins and EF1B (Bra008915), which is a translation elongation factor. Among the downregulated genes, there were three beta-1,3-glucanase genes, which have been reported to be induced by plant viruses and can accumulate in the vesicles and the cell wall to hydrolyze callose, resulting in the opening of the plasmodesmata (Epel, 2009). Although the correlation between proteome and transcriptome was low, there were still some valuable candidate genes. Bra006721 (nsLTP3) and Bra020322 (nsLTP4) present the same expression trend (upregulated) in the proteome and transcriptome. Bra025730 (pathogenesis-related thaumatin superfamily protein), Bra003273 (BETA-1, 3-GLUCANASE 3), Bra000315 (chitinase), Bra036259 (chitinase), and Bra004771 all showed an opposite expression trend (downregulated in the proteome, upregulated in the transcriptome) (Table 2). Based on the transcriptome and proteome data, we summarized the candidate genes and pathways related to TuMV infection on a preliminary network (Figure 7) that mainly focused on calcium signaling pathways, HSPs, WRKY transcription factors, and non-specific lipid transfer proteins.

## Discussion

### Plant Hormones Are Correlated to TuMV Resistance

Many viral diseases can lead to developmental abnormalities and aberrant phenotypes. This phenomenon may be induced by disruptions in hormone metabolism. The past decades witnessed an increase in the studies that demonstrated that viral infections can affect the production and distribution of many plant hormones such as auxin, cytokines, and gibberellins (Alazem and Lin, 2015). An interaction has been found between *Tobacco mosaic virus* (TMV) infection and the auxin-responsive pathway, which can influence the development of disease symptoms (Padmanabhan et al., 2005). Herein, we were able to obtain several auxin-responsive genes that may be related to this pathway. Moreover, since the SAUR family proteins were previously reported to be related to elongation (Gil et al., 1994), their downregulated expression may explain the growth stunting. Combined, during viral infection, a set of Aux/IAA proteins can interact with TuMV and disrupt their normal function. Since there was an antagonistic effector of SA-mediated defense signaling, the ABA levels were originally considered to be negatively correlated with disease resistance (Thaler and Bostock, 2004). However, a positive role played by ABA in increasing pathogen resistance was later revealed, and this was conferred by the induction of callose deposition at plasmodesmata, which can inhibit viral cell-to-cell movement (Mauch-Mani and Mauch, 2005). Here, we found five abscisic acid receptor PYR/PYL family genes which function at the apex of a passive regulator pathway controlling ABA signaling (Park et al., 2009).

### Several Resistant Genes Are Related to TuMV Infection

In the plant-pathogen interaction category, we found many resistance genes such as LRR related genes, WRKY genes, and transcription factor MYC genes. Leucine-rich repeat (LRR) proteins have been reported to be related to plant defense and in the resistance against various pathogens including fungi, bacteria, and viruses (Jones, 1997). Peng et al. (2013) cloned six members of the LRR II subfamily from non-heading Chinese cabbage and found that these genes were induced by TuMV infection. All the LRR genes identified here were flagellin-sensitive 2 (FLS2), which had structural and functional homologies with the other resistance genes and were also involved in the innate immune system (Gómez-Gómez and Boller, 2000). WRKY22 found in this study has been reported to be related to the MAPK pathway, which can confer resistance to both bacteria and fungi (Asai et al., 2002). Furthermore, WRKY33 was reported to be required for SA-dependent defense gene expression (Loake and Grant, 2007), thus, they may also be associated with TuMV resistance. MYC2 is an important bHLH transcription factor that can regulate the expression of JA-responsive genes (Zhai et al., 2013). Two MYC2 genes identified were both downregulated, consistent with the jasmonate ZIM domain-containing genes mentioned above. This may indicate that the JA pathway is involved in the TuMV infection process. Furthermore, two Calmodulin genes (CaMs; *Bra000214*, *Bra025438*) and five Calcium-like genes (CMLs; *Bra018143*, *Bra039271*, *Bra019503*, *Bra015727*, and *Bra003712*) were identified in this study. CaMs and CMLs are primary calcium sensors in all eukaryotes, and these have been reported to boost antiviral defense by causing rapid hypersensitive cell death either by forming necrotic lesions or by mediating RNA silencing (Yamakawa et al., 2001; McCormack and Braam, 2003; Perochon et al., 2011; Nakahara et al., 2012).

### Several Host Factors Are Associated With TuMV Multiplication

As obligate intracellular parasites, viruses need to recruit host factors for their infection cycle, and the lack of certain host factors can confer resistance to the host (Truniger and Aranda, 2009; Nagy and Pogany, 2012). In terms of the DEGs, we found several HSP70s. Aside from plant closteroviruses, viruses do not have their own HSP70 genes, although many have been reported to recruit host HSP70s to assist in virion assembly, replication, and cell-to-cell movement (Mine et al., 2012). All the HSP70s (*Bra001457*, *Bra035470*, and *Bra029761*) were upregulated, which means they may take part in certain fundamental phases in the plant virus life cycles. A DnaJ homolog gene (*Bra008607*), functioning as part of the Hsp70 chaperone and can inhibit the *Brome mosaic virus* (BMV) negative-strand RNA synthesis (Wang et al., 2004), was identified and was also upregulated. Additionally, HSP20s, which are small heat shock proteins (sHSPs) that have the same function as HSP70, were also identified. Both *OsHSP20* and *NbHSP20* have been demonstrated to interact with RNA-dependent RNA polymerase (RdRp) of *Rice stripe virus* (RSV) and their sub-location and expression were significantly influenced by RSV (Li et al., 2015). As the HSP family is an abundant and complex group (Wang et al., 2004), they may exercise different functions, which can explain their inconsistent expression levels. As for the DEPs, the translation elongation factor EF1B (Bra008915) had also been identified in *Nicotiana benthamiana* as a protein required for TMV infection (Hwang et al., 2013).

### Some nsLTPs May Improve Plant Virus Resistance

We discovered many candidate genes and proteins related to TuMV infection in the correlation analysis of the transcriptome and proteome. nsLTPs are ubiquitous in animals, plants, fungi, and bacteria (Bureau et al., 1996). Although the lipid transport protein should promote virus replication, because of its participation in the assembly of viral replication (de Castro et al., 2016), nsLTPs have also been documented to assist in intercellular lipid transport and in the formation and accumulation of wax, thereby protecting against pathogens (Kader, 1996; Cameron et al., 2006). In this study, we found that nsLTP3 (Bra020323 and Bra006721) and nsLTP4 (Bra020322) were also significantly upregulated at the proteome level. Currently, there have been many studies that have demonstrated the disease resistance function of nsLTPs, such as in the resistance to fungi and bacteria in barley, maize, spinach, radish, and *Arabidopsis* (Terras et al., 1992; Molina and García-Olmedo, 1993; Segura et al., 1993). Consistent with this study, Park et al. found that *Tobacco mosaic virus* (TMV-P0) can induce *CaLTP1* expression (Park et al., 2002). Sujuon et al. also found that the silencing of *CALTPI* and *CALTPII* by VIGS can increase susceptibility to *Xanthomonas campestris* pv. *vescatoria* as well as to *Pepper mosaic mottle virus* (PMMV), and that the overexpression of *CALTPI* and *CALTPII* can enhance resistance (Sarowar et al., 2009). Moreover, nsLTPs were suggested to be involved in long-distance signaling by functioning as the systemic mobile signal for systemic acquired resistance (SAR) in plants (Sarowar et al., 2009). Despite knowledge of several biological functions of nsLTPs, their precise function(s) remains elusive; however, based on recent studies, nsLTPs have been verified to be irrelevant to intracellular lipid transport, due to their intercellular localization and intercellular secretion. For example, Ace-AMP1, which is homologous to plant nsLTPs and is isolated from onion (*Allium cepa* L.) seed, cannot transport phospholipids but have antifungal activity (Cammue et al., 1995; Tassin et al., 1998). There were several nsLTPs whose lipid-binding activities were not correlated with their antifungal activities (Sun et al., 2008). Although direct evidence is lacking, based on the enhanced susceptibility observed in this study and those in the pepper CALTPI and CALTPII study and in the *Arabidopsis* DIR1 study, it is logical to speculate that nsLTPs might serve as transporters for mobile signals during TuMV infection.

### The Correlation Between Transcriptome and Proteome in TuMV Infection

To better elucidate the infection mechanism, we analyzed the correlation between the transcriptome and proteome by obtaining the PCC (Figure 6). Overall, the correlation between the transcriptome and proteome was rather low (*R* = −0.0211), and for those selected due to their significantly changed transcriptome and proteome, the same change trend was calculated to be *R* = 0.8000 and the opposite change trend was *R* = −0.4762. As previously reported, there was no necessary correlation between transcriptome and proteome, and normally the correlation was very low (Gygi et al., 1999; Foss et al., 2007; Ghazalpour et al., 2011). But there were exceptions, for example, an overall moderate correlation was obtained between transcriptome and proteome patterns of *Pseudomonas syringae* pv. *tomato* DC3000 *in planta* bacterial multi-omics (Nobori et al., 2020). Based on the fact that the transcriptome is the intermediate state of gene expression and that the proteome is the final state of gene expression, the two types of expression levels generally correspond to each other. On the other hand, organisms can also make full use of this network to regulate protein abundance through methods such as DNA methylation, RNA silencing, and protein degradation. Therefore, most data that lie between the transcriptome and proteome are relevant; furthermore, there exist a few genes that are not relevant due to these regulatory mechanisms. There are other abiotic factors that influence data correlation such as differences in the technical systems, differences between data types and methods, and the effects of proteins with high abundance.

## Data Availability Statement

Our RNA-sequencing data have been submitted to the GEO repository. The GEO accession number is GSE151932.

## Author Contributions

SL, CZ, LG, and XH conceived the study. SL, LG, and RZ completed the experiments. SL, CZ, and LG contributed to data analysis and manuscript preparation. CZ and XH participated in the planning of experiments and revising the manuscript. All the authors read and approved the final version of the manuscript.

## Conflict of Interest

The authors declare that the research was conducted in the absence of any commercial or financial relationships that could be construed as a potential conflict of interest.

## Footnotes

1 http://www.genomics.cn/index.php

2 https://rpkgs.datanovia.com/ggpubr/

3 http://brassicadb.org/brad/datasets/pub/Genomes/Brassica_rapa/V1.0/V1.5/

4 http://brassicadb.org/brad/datasets/pub/Genomes/Brassica_rapa/V1.0/V1.5/Brapa_sequence_v1.5.fa.gz

5 http://www.genome.jp/kegg/

6 http://www.ncbi.nlm.nih.gov/COG/
