# Supplementary figures and images for: Correlation Analysis of Expression Profile and Quantitative iTRAQ-LC-MS/MS Proteomics Reveals Resistance Mechanism Against TuMV in Chinese Cabbage (Brassica rapa ssp. pekinensis)
Source: Front Genet. 2020 Aug 20;11:963. doi: 10.3389/fgene.2020.00963 (PMC7469979; doi:10.3389/fgene.2020.00963)

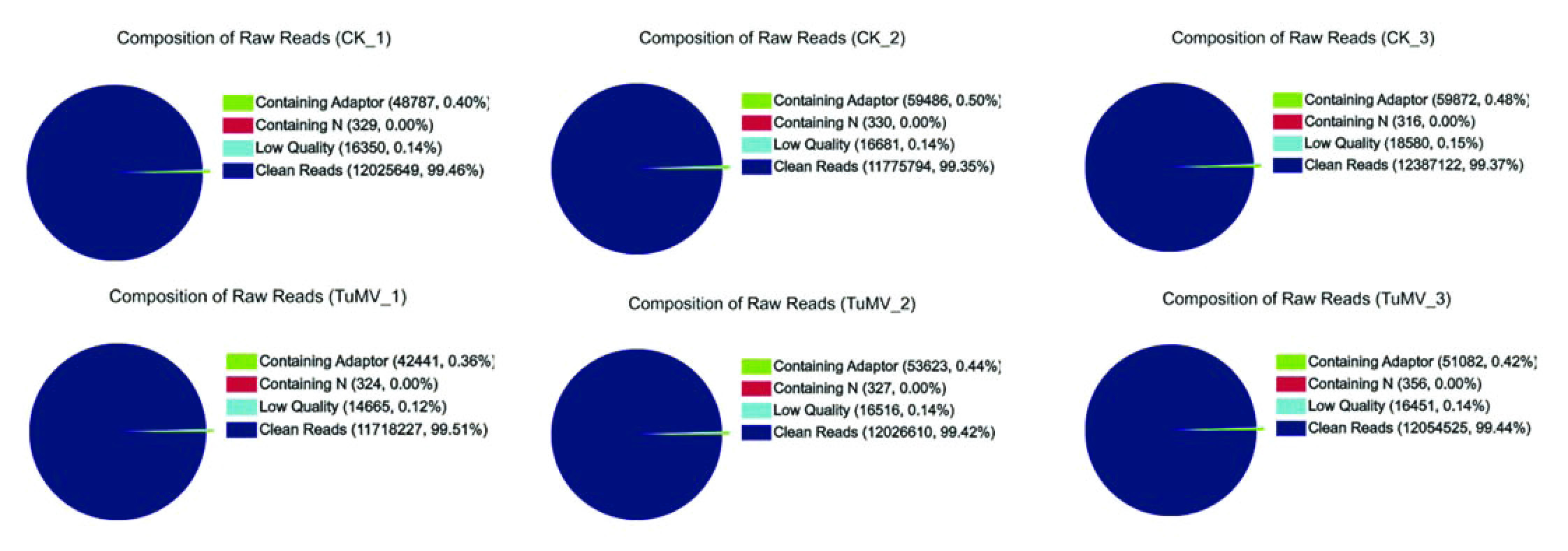

Supplement: Supplementary file 1 [file Image_1.JPEG]

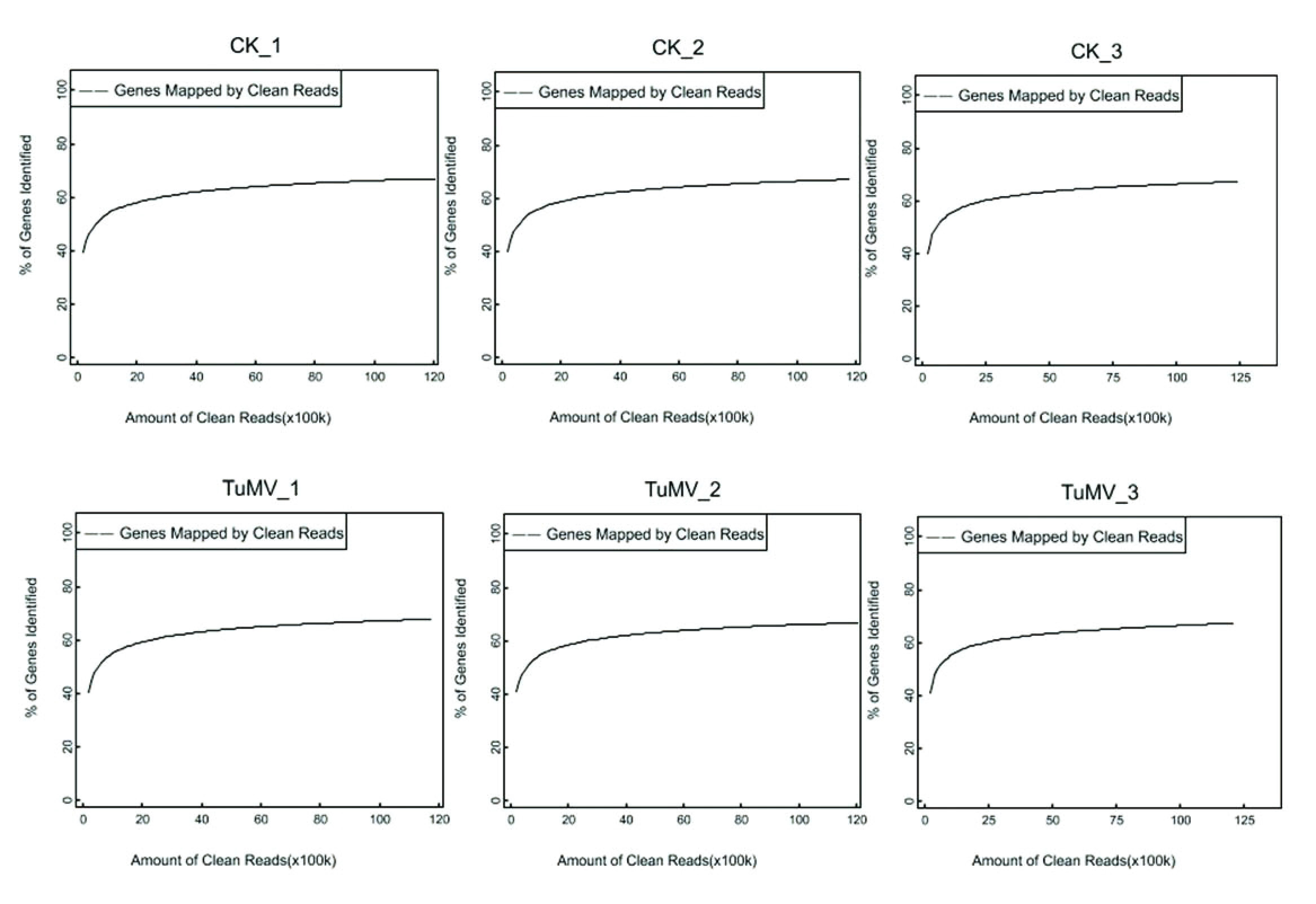

Supplement: Supplementary file 2 [file Image_2.JPEG]

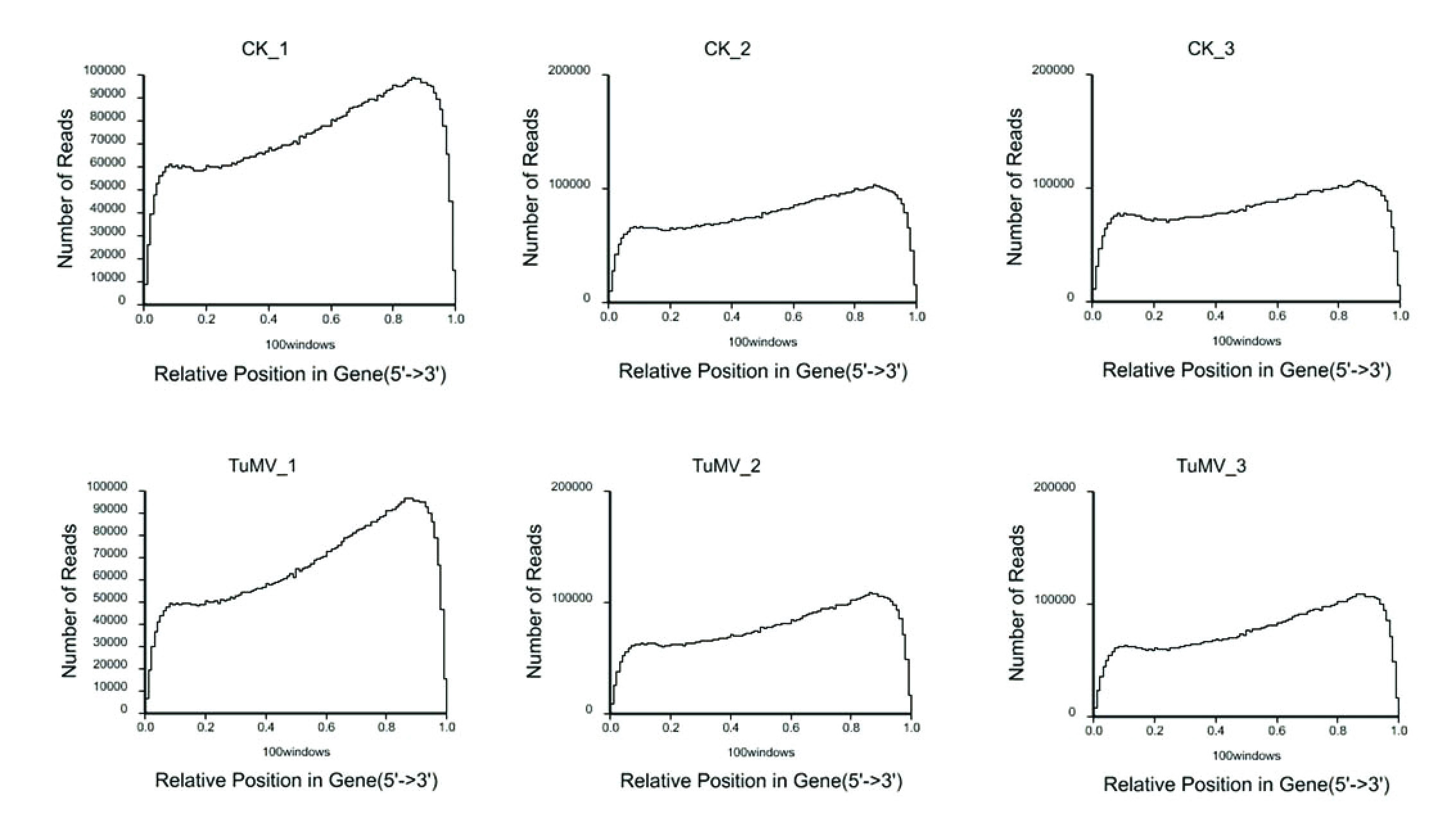

Supplement: Supplementary file 3 [file Image_3.JPEG]

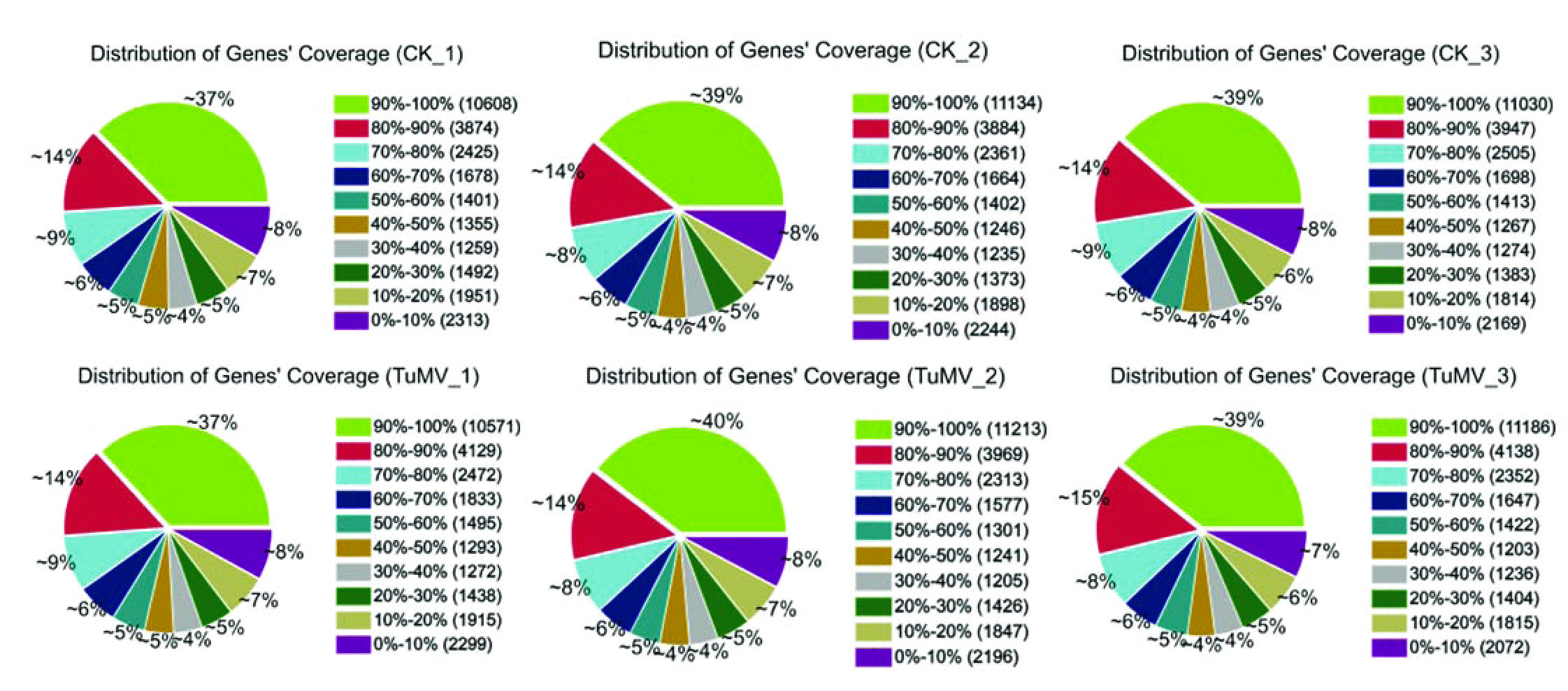

Supplement: Supplementary file 4 [file Image_4.JPEG]

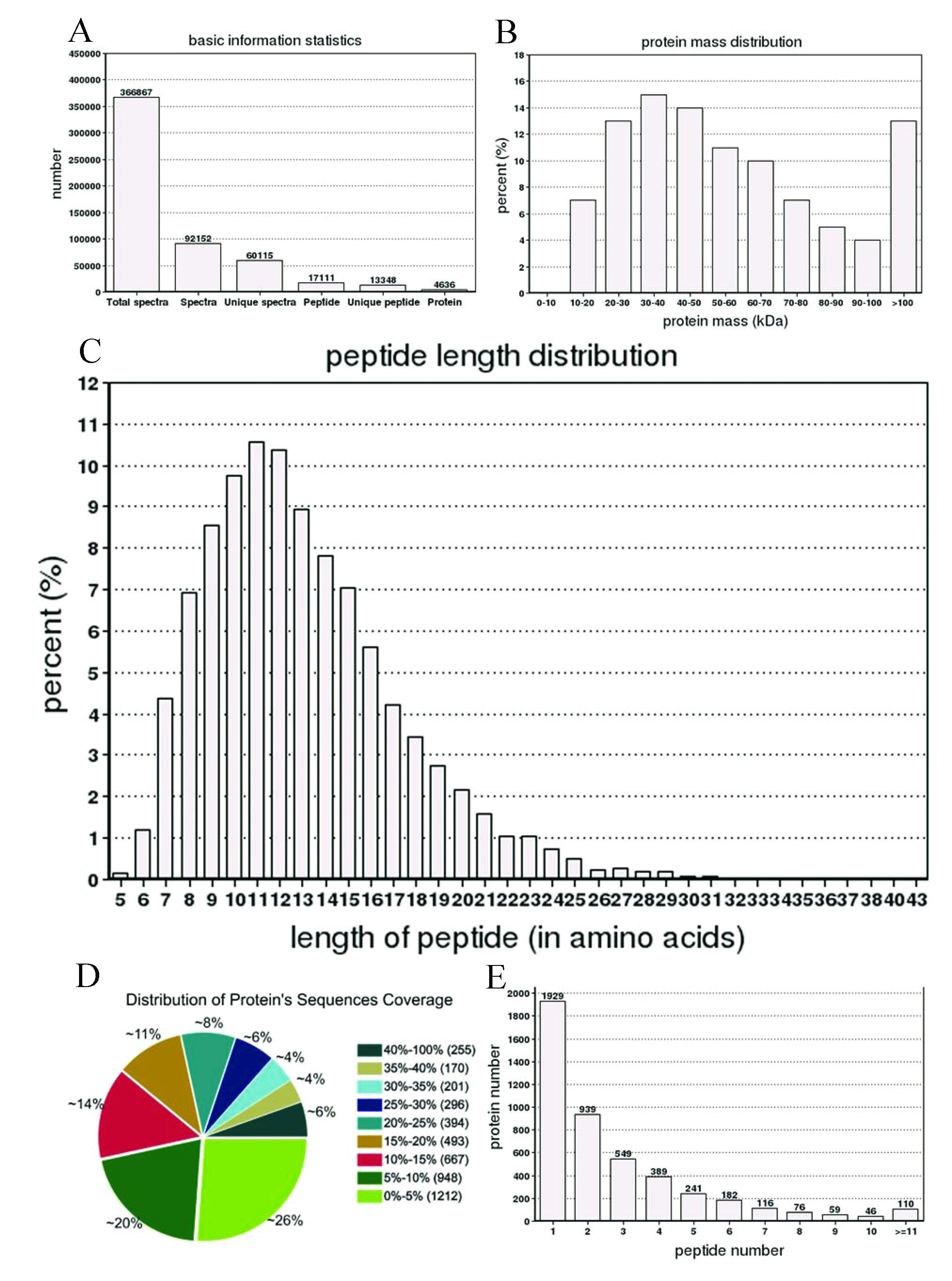

Supplement: Supplementary file 5 [file Image_5.JPEG]
